# An Overview of Risk Factors for Diabetic Foot Amputation: An Observational, Single-centre, Retrospective Cohort Study

**DOI:** 10.17925/EE.2023.19.1.85

**Published:** 2023-05-23

**Authors:** Burak Yuzuguldu, Bugra Zengin, Ilgin Yildirim Simsir, Sevki Cetinkalp

**Affiliations:** 1. Division of Pediatric Oncology, Dana-Farber Cancer Institute, Boston, MA, USA; 2. Division of Pediatric Surgery, Department of Surgery, Children’s Hospital of Pittsburgh, University of Pittsburgh School of Medicine, Pittsburgh, PA, USA; 3. Division of Endocrinology and Metabolism Disorders, Faculty of Medicine, Ege University, Izmir, Turkey

**Keywords:** Amputation, atherosclerosis, diabetic foot ulcer, haematocrit, hypertension, peripheral arterial disease, sepsis, thrombocytes, Wagner grading

## Abstract

Introduction: Not only are early detection and treatment of diabetic foot ulcers important, but also acknowledging potential risk factors for amputation gives clinicians a considerable advantage in preventing amputations. Amputations impact both healthcare services and the physical and mental health of patients. This study aimed to investigate the risk factors for amputation in patients with diabetic foot ulcers. Methods: The sample for this study was patients with diabetic foot ulcers who were treated by the diabetic foot council at our hospital between 2005 and 2020. A total of 32 risk factors for amputation were identified and investigated among 518 patients. Results: Our univariate analysis showed that 24 of 32 defined risk factors were statistically significant. In the multivariate analysis using the Cox regression model, seven risk factors remained statistically significant. The risk factors most significantly associated with amputation were Wagner grading, abnormal peripheral arteries, hypertension, high thrombocyte levels, low haematocrit levels, hypercholesterolaemia and male sex, respectively. The most common cause of death in patients with diabetes who have undergone amputation is cardiovascular disease, followed by sepsis. Conclusion: To enable optimum treatment of patients with diabetic foot ulcers it is important for physicians to be aware of the amputation risk factors, and thus avoid amputations. Correcting risk factors, using suitable footwear and routinely inspecting feet are crucial factors for preventing amputations in patients with diabetic foot ulcers.

Diabetic foot ulcers are one of the major complications in patients with diabetes mellitus (DM). In one study, the global prevalence of diabetic foot ulcers was 6.3%, and the prevalence of diabetic foot ulcers differed between North America (13.0%) and Europe (5.1%).^1^ Besides lowering quality of life, diabetic foot ulcers and diabetic foot amputations are a huge financial burden for both patients and healthcare systems. Jonasson et al. stated that 50–70% of all non-traumatic lower-l imb amputations are related to diabetes and its complications.^2^

In this study, we investigated the risk factors for amputation in patients with diabetic foot ulcers, to provide physicians with information on treating diabetic foot ulcers and preventing amputations (*Figure 1*). Preventing amputations due to diabetic foot ulcers is of paramount importance. Physicians should be aware of the risk factors for amputation in patients with diabetic foot ulcers to prevent worse outcomes. Thus, defining the risk factors predicting amputation is a vital medical approach.

## Methods

### Compliance with ethics

The study was approved by the Ege University Faculty of Medicine Ethics Committee with the decision dated 24 June 2020 and the approval number 20/6.1T/70. The study was conducted in accordance with the Helsinki Declaration principles, in full conformity to the laws and regulations of the Turkish Republic and full adherence to the principles described in the Good Clinical Practices. Informed consent was received from all patients.

### Study sample

The sample consisted of patients with diabetic foot ulcers who were treated by the diabetic foot council at our hospital between 2005 and 2020. The start date of the study was June 2005 and the end date was June 2020, with weekly follow-ups. The median follow-up time was 741 days. Patients were excluded if they had an unknown amputation status. Patients were divided into two groups: the amputation group and the non-amputation group. Patients with major (above-ankle) and minor (below-ankle) amputations were included in the amputation group; patients who had not undergone amputation were included in the nonamputation group. Patients who had undergone re-amputation were not included as an additional entry to the amputation group, only the first amputation of each patient was recorded. Seven patients who were scheduled to undergo the procedure but died before the scheduled date were included in the amputation group.

**Figure 1:**
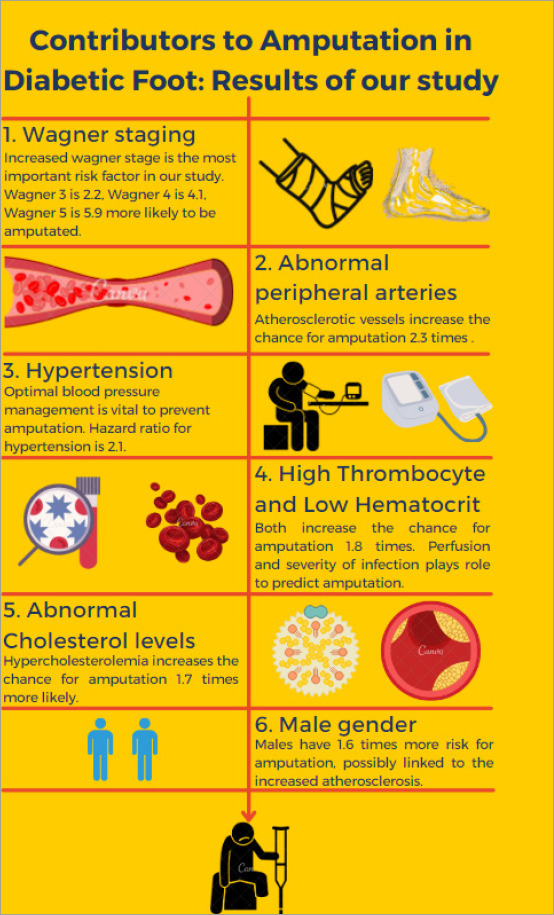
Significant contributing risk factors to amputation in patients with diabetic foot ulcers

### Methodology

The study design was an observational, single-centre, retrospective cohort study. Thirty-two risk factors for amputation in patients with diabetic foot ulcers were investigated.

The patients were grouped by factors that may have increased the chance of amputation; these factors included age, sex, DM type, DM duration, smoking status and several other factors.

Nephropathy was defined as either albuminuria or decreased glomerular filtration rate (GFR). Albuminuria was defined as an albumin–creatinine ratio >30 mg/g during a spot urine test. Nephropathy stages were determined by evaluating estimated GFRs and signs of nephropathy.

Signs of nephropathy were staged as follows, with all but stage 0 showing signs of nephropathy (such as albuminuria):

- Stage 0: GFR >60 mL/min/1.73 m^2^and no signs of nephropathy
- Stage 1: GFR >90 mL/min/1.73 m^2^
- Stage 2: GFR 60–89 mL/min/1.73 m^2^
- Stage 3A: GFR 45–59 mL/min/1.73 m^2^
- Stage 3B: GFR 30–44 mL/min/1.73 m^2^
- Stage 4: GFR 15–29 mL/min/1.73 m^2^
- Stage 5: GFR <15 mL/min/1.73 m^2^

Being on dialysis was a factor that was investigated, and patients who had previously received a kidney transplantation due to end-stage renal disease were excluded from the dialysis factor analysis. Retinopathy was defined by the presence of non-proliferative or proliferative diabetic retinopathy, based on ophthalmologic evaluation.

Hypertriglyceridaemia (HTG) was analysed via a blood test and was defined as triglyceride (TG) levels ≥150 mg/dL. Hypercholesterolaemia was defined as either low-density lipoprotein cholesterol (LDL-C ) levels ≥100 mg/dL or high-density lipoprotein cholesterol (HDL-C) levels <40 mg/dL for men, <50 mg/dL for women or total cholesterol levels ≥200 mg/dL. Hyperlipidaemia was defined as either the presence of hypercholesterolaemia or HTG.

Macrovascular complications were divided into three categories: peripheral arterial disease (PAD), cerebral artery disease (CAD) and coronary vascular disease (CVD). Symptomatic PAD was considered present when patients had an ankle-brachial index (ABI) of <0.9, imaging was consistent with PAD, or the presence of a stent or revascularization history. The contrast extremity imaging techniques used were computerized tomography angiography, magnetic resonance angiography or Doppler ultrasound. Normal ABI was considered 0.9–1.4; abnormal ABI was considered <0.9. Values >1.4 (incompressible) were excluded. Atherosclerotic peripheral arteries were defined as symptomatic or asymptomatic atherosclerosis findings by imaging or a history of PAD; normal peripheral arteries were defined as normal imaging or normal ABI without atherosclerosis or symptoms. CVD was considered positive when patients had a history of bypass, stent placement, positive angiography or myocardial infarction; CVD was considered negative when patients displayed normal exercise imaging, angiography or had a cardiology consultation with no history of coronary artery disease. The presence of CAD was evaluated by checking the patient's history for cerebrovascular accident or occluded cerebral artery in Doppler ultrasound. A normal Doppler, computerized tomography angiography of carotid arteries or magnetic resonance imaging diffusion without a history of cerebrovascular accident was considered as no CAD. Abdominal ultrasound was used to determine the presence and stage of hepatosteatosis.

The comorbidities, haematocrit, C-reactive protein (CRP), white blood cell (WBC) count, platelets, HbA1c, troglyceride and cholesterol levels, GFR, albumunuria and smoking status of patients were revealed by interviewing patients and using an electronic medical record (EMR). CRP levels were taken from the initial assessment of blood parameters. A CRP >0.5 mg/dL indicated infection, while a CRP >10 mg/dL indicated a severe bacterial infection.

Wagner grading was determined at ulcer diagnosis by the Wagner diabetic foot ulcer grade classification system using our hospital paperbased record system. Pathogenesis of ulcers was classified by the status of PAD or neuropathy assessed by EMR. The presence of only PAD was considered ischaemic, the presence of both PAD and neuropathy was considered neuroischaemic, and the absence of PAD was considered neuropathic.

Mortality status was determined using a national healthcare system, which showed whether the patient was alive or dead. If the patient died during hospitalization, the date and cause of death were assessed using EMR. Otherwise, this information was acquired from the caregivers of the patients. The last follow-u p date was 20 June 2020 for surviving patients. The amputation date was obtained using EMR.

### Statistical analysis

The IBM® SPSS® software (Armonk, NY, USA) was used to record the data and run the statistical analyses. First, we used mean and standard deviation for numeric values and numbers with percentages for categorical values. To determine if numeric values were parametric or non-parametric, the Kolmogorov–Smirnov test was used. As all variables were non-parametric, the Mann–Whitney U test was applied to verify whether the two groups were significantly different. Chi-squared and Fischer's exact tests were applied to the categorical data to compare the two groups.

Second, univariate analysis was investigated by using the Cox regression (proportional hazard) model to compare risk factors for amputations between groups. Time factor in Cox regression analysis was chosen as the time between diagnosis of a diabetic foot ulcer and either the last follow-up date for the non-amputation group or the amputation date for the amputation group. The primary outcome of the Cox regression analysis was the presence of amputation. Cut-off values for haematocrit, CRP, WBC count and platelet count were calculated through a receiver operating characteristic (ROC) curve. The cut-off value for albumin was defined by its normal range (<3.5 g/dL). Hypercholesterolaemia and hypertriglyceridaemia are defined by the guidelines.^3^

In the third step, the statistically significant risk factors found in the univariate analysis were applied in a multivariate analysis to eliminate confounding variables. The multivariate analysis was conducted using the backward elimination method. The Cox proportional hazard model was used to determine which factors remained statistically significant. An alpha level of 0.05 was set for significance.

The Kaplan–Meier method was used for assessing the mortality rates after amputations. For mortality analysis, the time factor was determined by the time between the amputation date and either last follow-up date or date of death. Statistical analysis was created using the Cox proportional hazard model to determine risk factors for death after amputation.

## Results

Initially, 523 patients were included in the study; however, five patients were excluded due to an unknown amputation status. Therefore, 518 patients were included in the study; of these, 311 had undergone amputations, and 207 patients had not.

**Figure 2:**
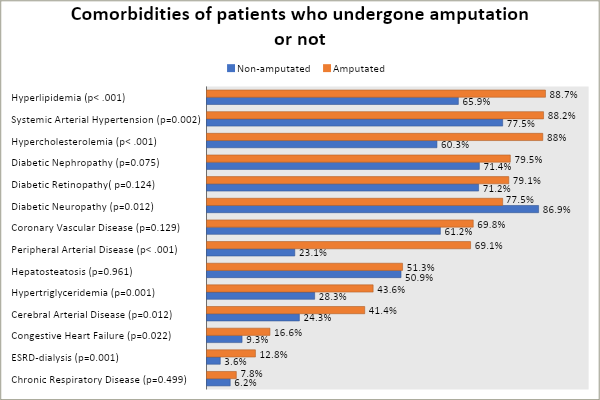
The comorbidities of patients who underwent amputation versus those who did not

The mean age was 63.4 ± 10.8 years; meanwhile, the mean age for patients who underwent amputation was 64.4 ± 10.7 years. There were 369 (71.2%) male patients and 149 (28.8%) female patients, with the male-to-female ratio being approximately 2.5:1. Thirty-five (6.8%) patients had type 1 DM, while 483 (93.2%) patients had type 2 DM. The mean diabetes duration was 16.9 ± 9.2 years. All 518 patients had a foot ulcer(s) at least once during the follow-up conducted by our multidisciplinary team, which consisted of nurses, residents and specialists in endocrinology, infectious disease and orthopaedic surgery. The mean and median follow-up periods were 33 months and 26 months, respectively; these values included survivors and patients who died during the study period. The mean and median times between diagnosis of foot ulcer and amputation in patients who had undergone amputation were 97 days and 60 days, respectively. The foot ulcers were grouped by the mechanism of ulcer formation: neuropathic, ischaemic or neuroischaemic. The pathogenesis was undetermined for 155 patients with diabetic foot ulcers, while 164 (45.2%) patients had neuropathic foot ulcers, 132 (36.4%) patients had neuroischaemic foot ulcers and 67 (18.5%) had ischaemic foot ulcers. The comorbidities of patients with or without amputation are shown in *Figure 2*.

*ESRD = end-stage renal disease*

For comparison of patients’ characteristics, demographic data and comorbidities, 34 elements were deemed potential risk factors (*Tables 1* *and* *2*). Of these, 25 potential risk factors were statistically significant for amputation age, sex, diabetes duration, neuropathy, albumin, decreased GFR, being on dialysis, TG level, HTG, hypercholesterolaemia, total cholesterol, HDL-C, hyperlipidaemia, atherosclerotic peripheral arteries, CAD, hypertension, congestive heart failure, CRP, haematocrit, WBC, platelet count, presence of infection, severe bacterial infection, gangrenous Wagner and ulcer pathogenesis (*Table 1* and *Table 2*).

In the univariate analysis among 518 cases, 32 potential risk factors were investigated using Cox proportional hazards, and 24 of them were statistically significant. The hazard ratios (HR), 95% confidence intervals (CI) and p-values are also included in *Table 3*. Prior to applying cut-off values, CRP level (HR 1.048, 95% CI 1.035–1.061; p<0.001), haematocrit level (HR 0.898, 95% CI 0.876–0.920; p<0.001), WBC count (HR 1.020, 95% CI 1.009–1.032; p<0.001) and platelet count (HR 1.003, 95% CI 1.002–1.004; p<0.001) were the statistically significant blood parameters in our univariate analysis. However, certain cut-off values in these parameters, calculated using a ROC curve, were more statistically significant than others (*Table 3*). Albumin (HR 0.434, 95% CI 0.361–0.523; p<0.001) is another blood parameter that was statistically significant in our univariate analysis; however, a cut-off value (3.5 g/dL) was defined and showed the most statistical significance. The results with those cutoff values are included in *Table 3*.

**Figure 3:**
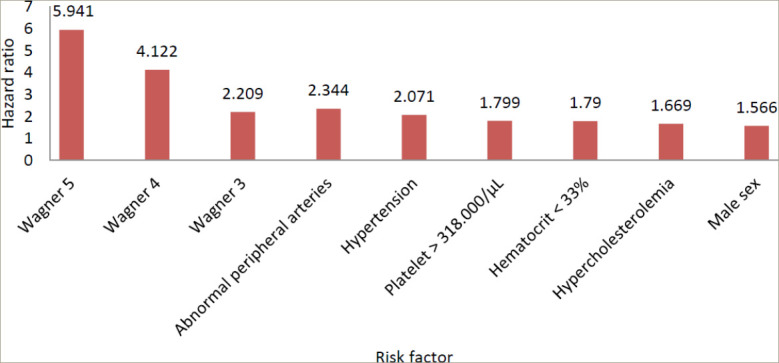
The statistically significant factors identified in the multivariate analysis

Of the 24 statistically significant factors found in the univariate analysis, the 15 factors with a p-value of <0.05 (CRP >2.12 mg/dL, sex, age, Wagner grade, hypertension, heart failure, hypercholesterolaemia, HTG, haematocrit <33%, WBC count >10.2×10^3^/µL, platelet count >318×10^3^/µL, atherosclerotic peripheral arteries, albumin <3.5 g/dL, GFR<30 mL/ min/1.73 m^2^ and pathogenesis) were applied in the multivariate analysis. By using the backward elimination method, seven of these risk factors remained statistically significant, and these were used to create the second and final multivariate analysis. We have provided the results of the second multivariate analysis. Wagner grading was the most important risk factor in predicting amputation, followed by atherosclerotic peripheral arteries, hypertension, increased platelet count, decreased haematocrit, hypercholesterolaemia and male sex, as shown in *Table 4* *and* *Figure 3*.

**Table 1:** Demographic data of patients with diabetic foot ulcers (numerical values)

Risk factors	Total (N=518)	Amputation (n=311)	Non-amputation (n=207)	p-value
Diabetes duration (mean ± SD)	16.9 ± 9.2	17.9 ± 9.4	15.3 ± 8.7	0.002
DM type (n [%])	518 (100.0)	311 (100.0)	207 (100.0)	0.478
Type 1	35 (6.8)	23 (7.4)	12 (5.8)	
Type 2	483 (93.2)	288 (92.6)	195 (94.2)	
Age (years) (mean ± SD)	63.4 ± 10.8	64.4 ± 10.7	61.8 ± 10.9	0.003
Sex (n [%])	518 (100.0)	311 (100.0)	207 (100.0)	0.002
Male	369 (71.2)	237 (76.2)	132 (63.8)	
Female	149 (28.8)	74 (23.8)	75 (36.2)	
Smoking (pack/year) (mean ± SD)	25.6 ± 33.8	28.5 ± 37.7	21.2 ± 26.2	0.217
Haemoglobin A1c (%) (mean ± SD)	8.7 ± 2.3	8.6 ± 2.1	8.7 ± 2.5	0.901
C-reactive protein (mg/dL) (mean ± SD)	6.7 ± 7.8	8.8 ± 7.9	4.0 ± 6.6	<0.001
Albumin (g/dL) (mean ± SD)	3.5 ± 0.7	3.3 ± 0.6	3.8 ± 0.7	<0.001
Haematocrit (%) (mean ± SD)	34.6 ± 5.8	32.8 ± 5.1	37.1 ± 5.8	<0.001
White blood cell count (10^3^/µL) (mean ± SD)	10.8 ± 5.8	11.8 ± 4.8	9.6 ± 6.7	<0.001
Platelet count (10^3^/µL) (mean ± SD)	324.7 ± 130.3	358.6 ± 133.7	281.1 ± 111.9	<0.001
Total cholesterol (mg/dL) (mean ± SD)	163.2 ± 46.3	160.3 ± 48.5	167.5 ± 42.6	0.044
Low-density lipoprotein cholesterol (mg/dL) (mean ± SD)	95.1 ± 37.1	94.7 ± 37.4	95.7 ± 36.7	0.926
High-density lipoprotein cholesterol (mg/dL) (mean ± SD)	38.5 ± 13.8	35.4 ± 13.7	43.2 ± 12.7	<0.001
Triglycerides (mg/dL) (mean ± SD)	150.9 ± 72.6	156.4 ± 72.5	142.6 ± 72.1	0.017

*DM = diabetes mellitus; SD = standard deviation.*

**Table 2:** Characteristics of patients with diabetic foot ulcers (categorical values)

Risk factors	Total n (%)	Amputation n (%)	Non-amputation n (%)	p-value
Gangrenous Wagner*	184/462 (39.8)	161/274 (58.8)	23/188 (12.2)	<0.001
Infection^†^	393/456 (86.2)	256/272 (94.1)	137/184 (74.5)	<0.001
Severe bacterial infection^‡^	113/455 (24.8)	93/271 (34.3)	20/184 (10.9)	<0.001
Pathogenesis of ulcer	363	244	119	<0.001
Neuropathic	164 (45.2)	76 (31.1)	88 (73.9)	
Neuroischaemic	132 (36.4)	115 (47.1)	17 (14.4)	
I schaemic	67 (18.5)	53 (21.7)	14 (11.8)	
Neuropathy	373/460 (81.1)	220/284 (77.5)	153/176 (86.9)	0.012
Retinopathy	229/300 (76.3)	155/196 (79.1)	74/104 (71.2)	0.124
Nephropathy	283/371 (76.3)	178/224 (79.5)	105/147 (71.4)	0.075
Glomerular filtration rate <60 mL/min/1.73 m^2^	157/483 (32.5)	102/282 (36.2)	55/201 (35.0)	0.042
Coronary vascular disease	224/333 (67.3)	164/235 (69.8)	60/98 (61.2)	0.129
Atherosclerotic peripheral arteries	292/373 (78.3)	235/256 (91.8)	57/117 (48.7)	<0.001
Cerebral arterial disease	81/226 (35.8)	63/152 (41.4)	18/74 (24.3)	0.012
Hypertension	403/480 (84.0)	255/289 (88.2)	148/191 (77.5)	0.002
Chronic respiratory disease	35/489 (7.2)	23/295 (7.8)	12/194 (6.2)	0.499
Congestive heart failure	67/488 (13.7)	49/295 (16.6)	18/193 (9.3)	0.022
Hepatosteatosis	111/217 (51.2)	82/160 (51.3)	29/57 (50.9)	0.961
Being on dialysis with end-stage renal disease	45/494 (9.1)	38/298 (12.8)	7/196 (3.6)	0.001
Hypercholesterolaemia	349/453 (77.0)	241/274 (88.0)	108/179 (60.3)	<0.001
Hypertriglyceridaemia	170/453 (37.5)	119/273 (43.6)	51/180 (28.3)	0.001
Hyperlipidaemia	361/453 (79.7)	243/274 (88.7)	118/179 (65.9)	<0.001

*Gangrenous Wagner defined as Wagner scores 4 and 5.

*^†^Defined as C-reactive protein >0.5 mg/dL.*

*^‡^Defined as C-reactive protein >10 mg/dL.*

**Table 3:** Results of univariate analysis with 518 cases by using Cox proportional hazards

Risk factor	Cox p-value	Hazard ratio	95.0% Confidence interval
Male sex	0.010	1.466	1.097–1.959
Diabetes type	0.504	1.202	0.701–2.061
Age	0.002	1.018	1.007–1.030
Smoking versus nonsmoking	0.040	1.004	1.000–1.008
Diabetes duration	0.060	1.014	0.999–1.028
Gangrenous* versus non-gangrenous Wagner grade	<0.001	4.467	3.378–5.907
Wagner 3 versus Wagner 0–2	<0.001	3.483	2.253–5.382
Wagner 4 versus Wagner 0–2	<0.001	7.306	5.003–10.668
Wagner 5 versus Wagner 0–2	<0.001	10.793	5.997–19.426
Nephropathy	0.051	1.958	0.999–1.958
Glomerular filtration rate <60 mL/min/1.73 m^2^	0.006	1.441	1.113–1.867
Glomerular filtration rate <30 mL/min/1.73 m^2^	0.003	1.670	1.189–2.344
Being on dialysis	0.006	1.700	1.166–2.480
Retinopathy	0.293	1.219	0.843–1.764
Neuropathy	<0.001	0.510	0.375–0.693
Atherosclerotic peripheral arteries (atherosclerosis and PAD versus non atherosclerosis)	<0.001	5.212	3.209–8.466
Cerebral arterial disease	0.005	1.660	1.168–2.360
Coronary vascular disease	0.360	1.155	0.848–1.573
Ischaemic versus neuropathic pathogenesis	<0.001	3.167	2.163–4.636
Neuroischaemic versus neuropathic pathogenesis	<0.001	2.492	1.810–3.430
Hypertension	0.001	1.968	1.300–2.980
Congestive heart failure	0.010	1.559	1.110–2.189
Hypercholesterolaemia	<0.001	3.501	2.323–5.276
Hypertriglyceridaemia	0.016	1.383	1.063–1.801
Chronic respiratory disease	0.428	1.209	0.756–1.932
Hepatosteatosis	0.473	1.131	0.807–1.585
High-density lipoprotein	<0.001	2.663	1.990–3.564
Presence of infection	<0.001	3.515	2.082–5.934
C-reactive protein >2.12 mg/dL	<0.001	3.708	2.744–5.010
Haematocrit <33.0%	<0.001	3.022	2.326–3.925
Albumin <3.5 g/dL	<0.001	2.717	2.079–3.553
White blood cell >10.2×10^3^/µL	<0.001	2.415	1.859–3.138
Platelet count >318×10^3^/µL	<0.001	2.291	1.764–2.975
Haemoglobin A1c	0.719	0.990	0.935–1.047

*Of the 518 patients included in the study, 311 had undergone amputation.*

*Gangrenous Wagner defined as Wagner scores 4 and 5.

**Table 4:** Statistically significant risk factors for amputation identified in the multivariate analysis

Risk factor	p-value	Hazard ratio	95.0% confidence interval
Wagner 5 versus Wagner 0–2	0.000	5.941	2.866–12.317
Wagner 4 versus Wagner 0–2	0.000	4.122	2.599–6.538
Wagner 3 versus Wagner 0–2	0.004	2.209	1.296–3.765
Atherosclerotic peripheral arteries	0.004	2.344	1.313–4.185
Hypertension	0.013	2.071	1.169–3.674
Platelet count >318×10^3^/µL	0.001	1.799	1.276–2.537
Haematocrit <33.0%	0.001	1.790	1.279–2.506
Hypercholesterolaemia	0.043	1.669	1.017–2.738
Male sex	0.019	1.566	1.075–2.282

*CI = confidence interval.*

Among 311 patients who had undergone amputation, 95 were dead at the follow-up date; the cause of death was known in 57 patients. The most common cause of death was cardiovascular (30 patients) followed by sepsis (15 patients), pneumonia (4 patients), respiratory causes (2 patients), fat embolism during follow-up (1 patient) and other causes (5 patients) (Suppl Figure 1). Of all patients with known causes of death in the amputation group, 85% died of cardiovascular or infective causes, demonstrating the importance of optimizing cardiovascular risk factors and infections in patients requiring amputation. Six of seven patients who died before the amputation operation died from sepsis, and the seventh patient died from cardiovascular complications, which displays the sensitivity of amputation timelines.

## Discussion

Our data showed that 60% of patients underwent amputation, which is a higher rate of amputation than that reported in other centres (6–43%), indicating the council’s tertiary care role in the healthcare system.^4^ In our study, multivariate analysis by Cox regression showed that seven factors are most significantly related to amputation in patients with diabetic foot ulcer: Wagner grade, atherosclerotic peripheral arteries, hypertension, thrombocytosis (a disorder in which your body produces too many platelets), decreased haematocrit, hypercholesterolaemia and male sex. Multiple factors were not included in the multivariate analysis. Of the risk factors found in the univariate analysis, 15 (CRP >2.12 mg/dL, sex, age, Wagner grade, hypertension, heart failure, hypercholesterolaemia, HTG, haematocrit <33.0%, WBC count >10.2×10^3^/µL, platelet count >318×10^3^/µL, atherosclerotic peripheral arteries, albumin <3.5 g/dL, GFR <30 mL/min/1.73 m^2^ and pathogenesis) were applied in multivariate analysis. Those 15 factors were selected if their p-values were <0.05 in the univariate analysis. CAD and abnormal ABI were not selected due to the low number of known cases. Smoking was not included as it was positively correlated in the univariate but negatively in the multivariate analysis, contradicting itself; compounds such as PAD and CVD are influenced by smoking, which would result in bias. Furthermore, patient interviews were subject to reporting bias. Neuropathy was not included as it forms pathogenesis. By using the backward likelihood ratio method, seven of these risk factors remained statistically significant. Multivariate analysis was created by using these seven variables.

**Figure 4:**
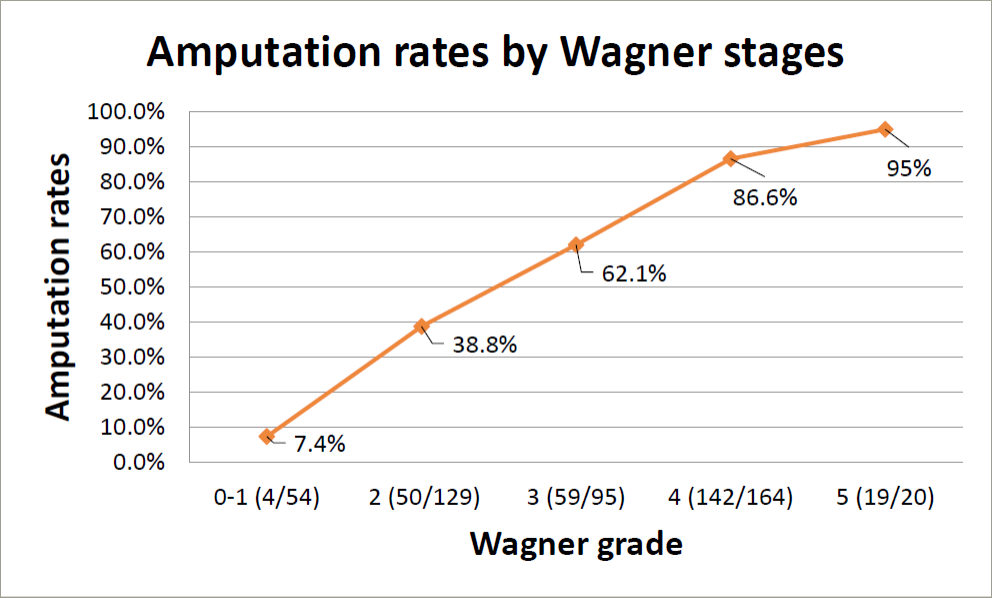
Amputation rates by Wagner stage

In our study, Wagner grade at diagnosis was the leading risk factor for amputation that enabled us to predict prognosis. Every unit increase in Wagner grade increased the chance of amputation by 2.233 times (95% CI 1.947–2.561; p<0.001). Similar results were found in previous studies that have demonstrated that Wagner grade directly correlated with an increased risk of amputation.^5,6^ Gul et al. stated similar amputation rates by Wagner grades to our findings, with their results being Wagner grade 1: 7.1%; Wagner grade 2: 18.6%; Wagner grade 3: 35.4%; Wagner grade 4: 66.6%; Wagner grade 5: 100.0%) (*Figure 4*).^7^ Uysal et al. found osteomyelitis (odds ratio [OR] 3.09; p<0.001) to be a positively correlated risk factor for amputation; similarly to our results, they found that having Wagner grade 3 increases the amputation risk by 3.5 times compared with Wagner grades 0-2 in the univariate analysis and 2.2 times in the multivariate analysis.^8^

*Wagner grades were determined at diagnosis of ulcers. Since we have reported classification at the time of diagnosis, some patients might delay treatments and progress to higher Wagner grades that necessitate amputation.*

In our study, patients with gangrenous Wagner grades (4 and 5) were 4.5 times more likely to require amputation than patients with non-gangrenous Wagner grades, thus demonstrating the importance of presence of gangrene in determining amputation risk. These findings are similar to those yielded in research from Nigeria (OR 5.953) and Ethiopia (OR 4.7).^9,10^ Lin et al. stated that having gangrene increased amputation risk by over 10 times, which makes it the most important risk factor in their study.^11^ In our multivariate analysis, diabetic foot ulcers in Wagner grades 3, 4 or 5 increased the amputation risk by 2.2, 4.1 and 5.9 times, respectively. Therefore, early diagnosis and treatment of diabetic foot ulcers in the lower Wagner grades are key in preventing amputation (Suppl. Figure 2).

Jensen et al. showed that one of three patients experienced undiagnosed atherosclerosis prior to the lower-extremity amputation.^12^ Our stepwise multivariate analysis showed that peripheral arterial diseases were the second most important predictor of amputation, following Wagner grade. One study found atherosclerosis and lower-l imb loss to be significantly associated.^13^ Kim et al. demonstrated that carotid intima media thickness, an atherosclerosis predictor, significantly correlated with amputation rates in patients with diabetic foot ulcers.^13^ There are several risk factors for atherosclerosis, and new risk factors for atherosclerosis continue to be discovered. The major atherosclerosis risk factors in this study included age, sex, smoking, hyperlipidaemia, HTG, hypercholesterolaemia and hypertension.^14^ All were significantly associated with amputation in the univariate analysis; however, only hypertension, hypercholesterolaemia and sex remained significant in our multivariate analysis. Even though macrovascular complications resulting from atherosclerosis, such as CAD and PAD, were significantly associated with amputation, CVD was similar in patients despite amputation status with no significant difference between groups.

In our study, smoking and age both demonstrated statistical significance in univariate analysis but not in our multivariate analysis. Smoking wasn’t included in the multivariate analysis. Smoking one pack of tobacco per year was found to increase amputation risk by 0.4% in our study. Other research studies have shown similar results (HR 1.19–1.65).^11,15–17^ Although age was statistically significant in our univariate analysis (HR 1.018; p=0.002), it became non-significant in the multivariate analysis. This may be because, although smoking and age are correlated with atherosclerosis and the resulting amputation, they are not as strong predictors as other components in the multivariate analysis.

In our univariate analysis, symptomatic PAD increased amputation chance by 2.9 times (95% CI 2.151–3.876; p<0.001). Several studies have highlighted the correlation between PAD and amputation rates, thus reflecting our findings. Perng et al. stated that having PAD increases the likelihood of amputation by around 3.5 times and that PAD was the most important risk factor in their study.^18^ Pemayun et al.,^19^ Sen et al.,^15^ Ugwu et al.,^9^ Sayiner et al.^16^ and Ndip et al.^20^ found the following ORs: 2.11, 2.35, 2.8, 3.35 and 3.8, respectively. Ugwu et al., Sayiner et al., Perng et al., Ndip et al. and Pemayun et al. found the following ORs in their multivariate analysis: 2.8, 2.97, 3.196, 4.1 and 12.97, respectively.^9,16,18–20^ Peripheral vascular calcification with or without PAD increased the chance of amputation by 5.2 times compared with normal peripheral arteries in our univariate analysis. In our multivariate analysis, we included atherosclerotic peripheral arteries, with or without symptomatic PAD, as opposed to symptomatic PAD alone, for better analysis. In the multivariate analysis, having atherosclerosis in peripheral arteries increased the amputation risk by >2.3 times. Simsir et al. found that fetuin-alpha levels correlated with the amputation level and vascular calcification, elucidating one of the molecular factors in predicting amputation.^21^ We did not measure fibrinogen and homocysteine; however, physicians should remain alert for asymptomatic atherosclerosis in peripheral arteries to prevent worse outcomes. sen

Our multivariate analysis showed that hypertension was the third most important risk factor for amputation, increasing the amputation risk over two-fold. Hypertension significantly affects both macrovascular and microvascular complications in diabetes. While being one of the most important risk factors for nephropathy, hypertension also severely disrupts the vascular endothelium, increases atherosclerosis and causes end-organ damage.^22,23^ In our univariate analysis, almost twice as many patients with hypertension underwent amputation as those without hypertension. Previous studies in the literature have also found hypertension to be a risk factor in their univariate analysis, (with ORs ranging from 1.19–3.15).^11,16,19,24,25^

Infection severity also plays a role in the prognosis of patients with diabetic foot ulcer. There have been several studies to find blood markers that can inform infection severity when making the decision for amputation.^26–29^ In our univariate analysis, levels of CRP >2.12 mg/dL, platelet counts >318×10^3^/µL, WBC count >10.2×10^3^/µL and blood albumin levels <3.5 g/ dL were important markers of infection severity predicting amputation. Several studies also yielded similar results.^9,11,26,30,31^ However, when included in our multivariate analysis, only increased platelet levels showed statistical significance. Although infection is the major cause of secondary thrombocytosis, there are limited studies showing the relationship between thrombocytosis and diabetic foot amputation. In our univariate analysis, platelet levels >318×10^3^/µL increased the amputation risk by 2.3 times; in our multivariate analysis, it increased the amputation risk by 1.8 times. This increase may be caused by disease-specific factors. One study showed that thrombocytosis is most commonly caused by soft-tissue infections and predisposes patients to methicillin-resistant *Staphylococcus aureus* (MRSA) infections.^32^ Physicians frequently encounter MRSA infection in patients with diabetic foot ulcers, and MRSA infections are associated with a poor prognosis in patients with diabetic foot ulcers.^32–34^ However, there is still not enough information about this relationship in the literature. Providers should properly treat the underlying infection to prevent disastrous outcomes.

The next significant risk factor was haematocrit. Severe infection causes low haematocrit level; as haematocrit decreases, tissue perfusion becomes inefficient and affects outcomes. However, the relationship between haematocrit and amputation has not been sufficiently investigated. In a study in Singapore, haemoglobin levels ≤10 g/dL increased amputation frequency by five times, supporting our results.^35^ In our study, haematocrit percentage was inversely associated with amputation (HR 0.898, 95% CI 0.876–0.920; p<0.001) when the cut-off value was set to 33.0% using a ROC curve. In the univariate analysis, haematocrit levels <33.0% resulted in amputations being three times more likely in our univariate analysis (HR 3.022, 95% CI 2.326-3.925; p<0.001), and haematocrit remained significant in the multivariate analysis with the HR of 1.79 (95% CI 1.279–2.506; p=0.001). When treating infection, physicians should also be alert to the presence of anaemia to decrease the likelihood of undesirable consequences.

Currently, the role of lipid abnormalities in diabetic foot amputation is disputed, as available data are limited. Some studies claim that lipid abnormalities significantly impact diabetic foot ulcer prognosis, while others even suggest that hyperlipidaemia is negatively correlated with major amputation, which could be due to surveillance bias.^36,37^ Our study showed that hypercholesterolaemia and HTG were both significantly associated with amputation in the univariate analysis. In our study, patients with TG levels <150 mg/dL were 1.383 times more likely to require amputation, contradicting several studies hypothesizing that increased TG levels might have a protective effect on amputation.^38,39^ In a study in India, hypertriglyceridaemia increased amputation rates, supporting our findings.^37^ However, when included in our stepwise analysis, hypertriglyceridaemia lost its significance, leaving hypercholesterolaemia as an independent risk factor for amputation (HR 1.669, 95% CI 1.017–2.738; p=0.043). This could indicate that amputation risk is determined more by disrupted cholesterol levels than by TG levels, possibly due to the chronic nature of cholesterol level alterations.

The role of LDL-C and total cholesterol on amputation remains disputed.^19,37^When HDL-C was analysed as a categorical variable, HDL-C <40 mg/dL became significantly associated with amputation (HR 2.663, 95% CI 1.990–3.564; p<0.001). This finding reflects the findings by Ikura et al., showing the critical role played by HDL-C in predicting amputation compared with LDL-C and total cholesterol (HR 2.96, 95% CI 1.75–5.05; p<0.001).^40^ Consequently, appropriately treating patients for underlying cholesterol abnormalities is vital in helping to prevent amputations. Apolipoprotein B levels correlate with LDL-C and thus reflect the true picture of LDL-C, since it shows the LDL-C particles;^41^ however, our study is apolipoprotein B levels were not measured consistently during the study course.^41^

Sex was another predictor for amputation risk, with male patients having an increased likelihood of amputation by almost 1.5 times versus female patients (HR 1.466, 95% CI 1.097–1.959; p=0.010). In our stepwise analysis, male sex remained a significant risk factor of amputation (HR 1.566, 95% CI 1.075–2.282; p=0.019). The literature also suggests that being male is a predisposing factor for amputation (HR 1.3–2.8);^11,15,19,36^ however, a study conducted in Nigeria found that being male is not statistically significant predictor for amputation.^9^

Increased haemoglobin A1c (HbA1c) is frequently defined as an independent risk factor for amputation.^19,38,42,43^ Two landmark trials, the United Kingdom Prospective Diabetes Study and the Diabetes Control and Complications Trial, found that glycaemic control, especially in the early years of diabetes diagnosis, significantly improved diabetic complications.^44,45^ Controlled glucose levels in the first year of diagnosis has shown better outcomes in terms of microvascular complications up to 10 years.^46–48^ Recent research in Chicago showed that HbA1c levels <6.5% in the first year of diagnosis decreased both macrovascular and microvascular complications, which can be explained by the 'legacy effect'.^49,50^ According to the legacy effect, metabolic memory formed by consistently increased glucose levels in the early years of diagnosis adversely affects later outcomes. However, similarly to a meta-analysis that found that HbA1c levels were non-significant predictors of amputation,^11^ our study found that patients who underwent amputation and those who did not had similar HbA1c levels, which contradicts the above findings. We believe that this result might be confounded by the DM duration, as impaired management of early DM can cause disastrous outcomes (as stated above), even after similar glycaemic control later in the diagnosis, which we will call 'bad legacy of the metabolic memory'.^49^

As such, patients with a DM duration ≥10 years were then excluded to assess the effect of HbA1c on the amputation risk. HbA1c levels ≥7% increased amputation rates significantly in 93 of 104 patients with a DM duration of <10 years (HR 2.089, 95% CI 1.026–4.254; p=0.042; Suppl. Table 1); however, HbA1c levels were non-significant when including all patients with or without 10 years of diabetes. This finding is consistent with the hypothesis that early, strict and individualized glycaemic control has better outcomes for amputation in patients with diabetic foot ulcer. Patients with a DM duration ≥10 years may still have the 'bad legacy of the metabolic memories' formed in their early years, even after improving their glycaemic control.

There was no association between diabetes duration and amputation in our study, and most studies in the literature found similar results.^15,19,51,52^ Metabolic disease and its complications often precede diabetes diagnosis, especially in the in limited resourced countries (up to 50%);^53^ therefore, we conclude that DM duration is not an effective predictor of amputation.

In our study, GFR levels <60 mL/min/1.73 m^2^ (HR 1.441, 95% CI 1.113–1.867; p=0.006), <30 mL/min/1.73 m^2^ (HR 1.670, 95% CI 1.189–2.344; p=0.003) and being on dialysis with end-stage renal disease (HR 1.700; 95% CI 1.166–2.480; p=0.006) showed a positive correlation with amputation, while the presence of nephropathy (p=0.051) or albuminuria (p=0.14) were not statistically significant, which suggests that amputation risk increases in later stages of kidney disease when the GFR is worsened by nephropathy. The literature indicates that when GFR is 30–60 mL/ min/1.73 m^2^, the likelihood of amputation increases by almost two times versus GFR >60 mL/min/1.73 m^2^.^54^ Dialysis was also an independent risk factor for amputation.^20^ However, in our multivariate analysis, dialysis and decreased GFR lost their significance. The cause of this could be other atherosclerotic risk factors also affecting the risk of amputation, and the mechanism of chronic kidney disease being mostly atherosclerosis related, which was a factor also included in our multivariate analysis (peripheral arterial diseases, hypercholesterolaemia).^55^ Therefore, it is crucial to treat hyperglycaemia and hypertension to slow down nephropathy in the early phase and avoid late-stage nephropathy and the resulting accelerated atherosclerosis.

### Limitations

Our data were obtained from a single centre and incidences vary by centre. Furthermore, since our university is specialized in tertiary care, patients may be subjected to referral (admission rate) bias. Some percentages, such as the amputation rate, could be higher than the population average. Changing the providers during the 15-year time interval makes the study vulnerable to provider bias. Smoking pack/ year was estimated based on patient interviews and thus was subjected to reporting bias. Ulcer classification was based on neuropathy and/ or PAD and was decided based on EMR, which caused difficulty when determining neuropathy status in patients. Charcot arthropathy, osteomyelitis incidence, revision amputations, and data to classify aetiology of foot ulcers were unavailable in 155 patients. The study was retrospective and so causation cannot be assumed, only associations. Apolipoprotein B levels were not measured consistently during the study and therefore it was not feasible to reflect the true picture in terms of LDL-C.

## Conclusion

Diabetic foot ulcer is a challenging disease that is difficult to manage without an organized approach and multidisciplinary team. Timely amputation may even save lives when it is an unavoidable option; however, it is still a tremendous burden on patients’ lives. Wagner grade is the most important factor when it comes to deciding whether to amputate or not. Frequent foot inspections by the patient, their caregivers and healthcare professionals, the use of suitable footwear, and quick diagnosis and treatment of lesions are crucial to avoid amputations. Being aware of gangrene, avoiding higher Wagner grades with early detection and treatment, staying alert for asymptomatic PAD, reducing atherosclerotic risk factors by treating dyslipidaemia, optimizing blood pressure and properly controlling underlying infection are key to managing diabetic foot ulcers and avoiding minor or major amputations. Haematocrit is a crucial indicator of prognosis. Strict and individualized glycaemic control is especially important in the early years of diabetes diagnosis to prevent amputations. Managing cardiovascular risk factors and treating infection is vital, not only in preventing amputation, but also the prevention of the mortality during the postoperative period when amputation was required.
